# Supplementary material for: Biomechanical Variability and Usability of a Novel Customizable Fracture Fixation Technique
Source: Bioengineering (Basel). 2023 Sep 29;10(10):1146. doi: 10.3390/bioengineering10101146 (PMC10604275; doi:10.3390/bioengineering10101146)
Supplement: Supplementary file 1 [file bioengineering-10-01146-s001.zip › bioengineering-2616626-supplementary.pdf]

AdhFix Instruction Manual

Introduction

AdhFix is a light-cured composite that is anchored to the bone via conventional metal screws. The process of application is outlined in a stepwise manner in the following section. The goal is to achieve a uniform patch of dimensions of 25 (L) x 6 (W) x 1.5-2 mm (H) as shown on the example in front of you. The screws are pre-inserted, and the dimensions of the patch have been outlined on the synthetic bone. The composite is hardened with a Bluephase® light source using the Turbo mode. It is important that the hardening procedure is done thoroughly as illustrated in the stepwise guide. Each surface area should be hardened with two light-pulses each with a five second duration. For the first pulse, keep the light source close (2-3 mm) to the composite without touching it. For the second pulse, press the light source against the composite surface. Remember to wear UV-protective glasses when operating the light source. Tinfoil paper is to be left over the syringe to avoid unwanted curing of the composite within the syringe. The tip of the syringe should also be in tinfoil when not in use.

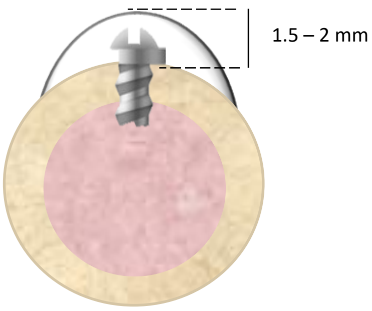

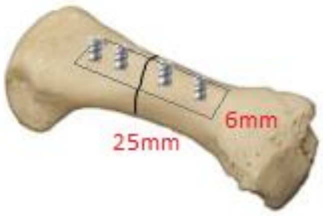

AdhFix Application Procedure

STEPS	DESCRIPTION	PICTURES
Step 1	Start the timer	
Step 2	***Please note:*** *Do Step 2, Step 3* ***Error! Reference source not found.*** *and Step 4 for two screws at a time.*<br><br>Place AdhFix composite around the shaft of each screw. Make sure there is composite around	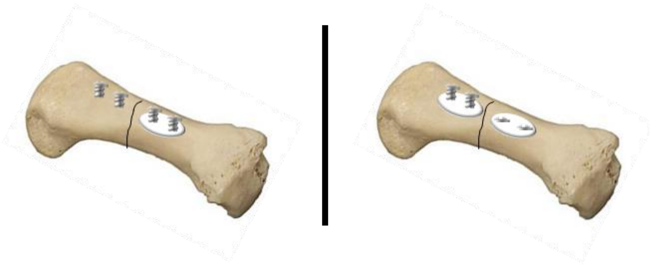

	the whole screw.	
Step 3	Insert the screws fully, do not force the screw deeper when resistance from the screw head coming in contact with bone is felt (Two-finger tight).	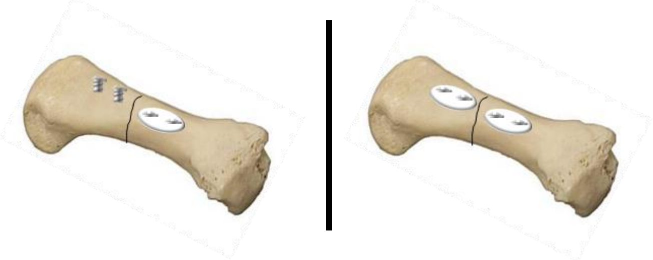
Step 4	Harden the composite around each screw head with two 5 sec pulses using the Bluephase lamp on Turbo mode (5 sec is automated)<br><br>***Please note 1:*** *Redo Step 2, Step 3* ***Error! Reference source not found.*** *and Step 4 for the next two screws.*<br><br>***Please note 2:*** *It is important to keep the Bluephase lamp at the same spot and as close to the composite surface as possible (2-3 mm) for an entire pulse time.*	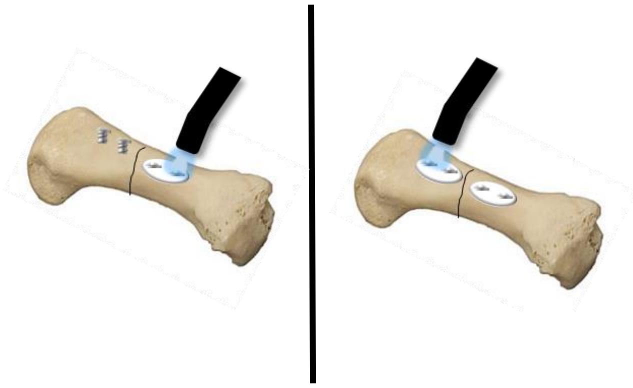
Step 5	Align the bone fragments and apply composite across the fracture gap to join the previously hardened composite patches on each side of the fracture.<br><br>Harden the added composite with two 5 s pulses per surface area using the Bluephase lamp on Turbo mode.	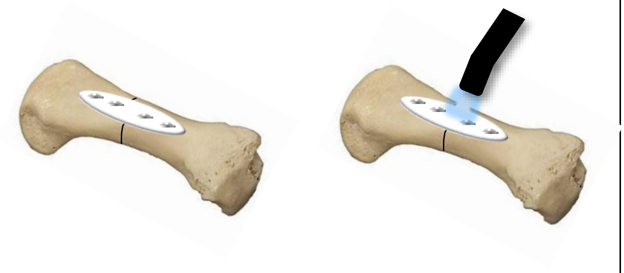

Step 6	Apply composite to the entire patch covering the screws to build up the width and length of the of the plate according to the markings. Use the plastic spatula to align the sides if needed.<br><br>***Optionally:*** *Pre-harden the added composite by sweeping the Bluephase lamp over the entire implant while doing two 5 s pulses on Turbo mode*.<br><br>Harden the entire implant with two 5 s pulses per surface area using the Bluephase lamp on Turbo mode.<br><br>***Please note:*** *It is important to overlap each spot that you shine light on to ensure complete curing of the composite. In this example 12 spots are necessary to cover the whole composite implant, leading to 24 pulses.*	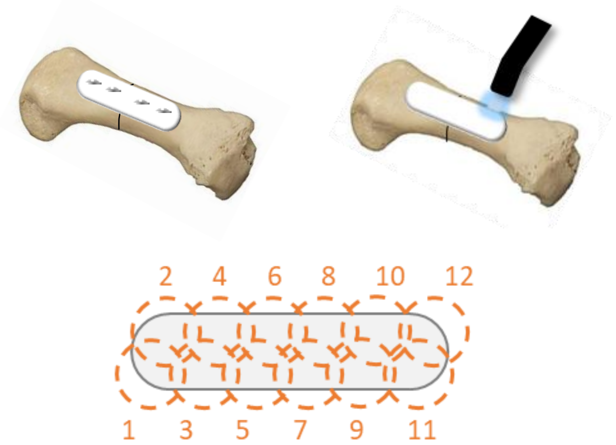
Step 7	Cover the composite plate and screw heads with a second layer of composite. Harden the added composite in the same way as in **Error! Reference source not found.**. The thickness of the final hardened implant should reach 1.5 to 2 mm.	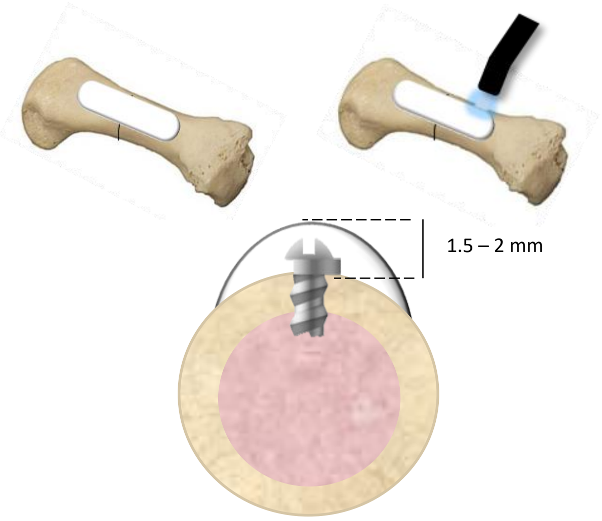
Step 8	The osteosynthesis is now complete.<br>End the timer	
